# Fast Approximate Quadratic Programming for Graph Matching

**DOI:** 10.1371/journal.pone.0121002

**Published:** 2015-04-17

**Authors:** Joshua T. Vogelstein, John M. Conroy, Vince Lyzinski, Louis J. Podrazik, Steven G. Kratzer, Eric T. Harley, Donniell E. Fishkind, R. Jacob Vogelstein, Carey E. Priebe

**Affiliations:** 1 Department of Biomedical Engineering, Johns Hopkins University, Baltimore, MD, USA; 2 Institute for Defense Analyses, Center for Computing Sciences, Bowie, MD, USA; 3 Johns Hopkins University Human Language Technology Center of Excellence, Baltimore, MD, USA; 4 Department of Applied Mathematics and Statistics, Johns Hopkins University, Baltimore, MD, USA; 5 Johns Hopkins University Applied Physics Laboratory, Laurel, MD, USA; Manchester University, UNITED KINGDOM

## Abstract

Quadratic assignment problems arise in a wide variety of domains, spanning operations research, graph theory, computer vision, and neuroscience, to name a few. The graph matching problem is a special case of the quadratic assignment problem, and graph matching is increasingly important as graph-valued data is becoming more prominent. With the aim of efficiently and accurately matching the large graphs common in big data, we present our graph matching algorithm, the Fast Approximate Quadratic assignment algorithm. We empirically demonstrate that our algorithm is faster and achieves a lower objective value on over 80% of the QAPLIB benchmark library, compared with the previous state-of-the-art. Applying our algorithm to our motivating example, matching C. elegans connectomes (brain-graphs), we find that it efficiently achieves performance.

## 1 Introduction

In its most general form, the graph matching problem (GMP)—finding an alignment of the vertices of two graphs which minimizes the number of induced edge disagreements—is equivalent to a quadratic assignment problem (QAP) [1]. QAPs were first devised by Koopmans and Beckmann in 1957 to solve a ubiquitous problem in distributed resource allocation [2], and many important problems in combinatorial optimization (for example, the “traveling salesman problem,” and the GMP) have been shown to be specialized QAPs. While QAPs are known to be **NP**-hard in general [3], they are widely applicable and there is a large literature devoted to their approximation and formulation; see [4] for a comprehensive literature survey. In casting the GMP as a QAP, we bring to bear a host of existing optimization theoretic tools and methodologies for addressing graph matching: Frank-Wolfe [5], gradient-descent [6], etc.

Graph matching has applications in a wide variety of disciplines, spanning computer vision, image analysis, pattern recognition, and neuroscience; see [7] for a comprehensive survey of the graph matching literature. We are motivated by applications in “connectomics,” an emerging discipline within neuroscience devoted to the study of brain-graphs, where vertices represent (collections of) neurons and edges represent connections between them [8, 9]. Analyzing connectomes is an important step for many neurobiological inference tasks. For example, it is becoming increasingly popular to diagnose neurological diseases via comparing brain images [10]. To date, however, these comparisons have largely rested on anatomical (e.g., shape) comparisons, not graph (e.g., structural) comparisons. This is despite the widely held doctrine that many psychiatric disorders are fundamentally “connectopathies,” i.e. disorders of the connections of the brain [11–14]. Thus, available tests for connectopic explanations of psychiatric disorders hinge on first choosing particular graph invariants to compare across populations, rather than comparing the graphs’ structure directly. Yet, recent results suggest that the graph invariant approach to classifying is both theoretically and practically inferior to comparing whole graphs via matching [15].

Part of the reason for the lack of publications that structurally compare brain-graphs is that existing algorithms for matching very large graphs are largely ineffective, often sacrificing accuracy for efficiency. Indeed, available human connectomes have 𝒪(10^6^) vertices and 𝒪(10^8^) edges, and building them leverages state-of-the-art advances in DT-MRI imagery, big data processing and computer vision [16]. Contrast this with the fact that the human brain consists of approximately 86 billion neurons [17]. At the other end of the spectrum, the small hermaphroditic *Caenorhabditis elegans* worm (*C. elegans*) has only 302 neurons, with a fully mapped connectome. Consequently, these graphs demand GM algorithms that both accurately match small–to–moderately sized graphs and also scale to match very large graphs.

When matching these large connectome graphs (and, more broadly, the large graphs common in big data [18]), we necessarily face an accuracy/efficiency trade-off when approximately solving these GMPs: slower algorithms could achieve better performance given more time (at the extreme, exhaustive search algorithms clearly have optimal performance). Our algorithm—the Fast Approximate QAP (FAQ) algorithm for approximate GM—achieves the best available trade-off between accuracy and efficiency, outperforming the existing state-of-the-art in both accuracy and efficiency on a large proportion of QAP benchmarks and biologically inspired network matching problems.

The remainder of this paper is organized as follows. Section 2.1 formally defines the QAP and a relaxation thereof that we will operate under. Section 2.3 defines graph matching, and explains how it can be posed as a QAP.

Section 3 describes the FAQ algorithm. Section 4 provides a number of theoretical and empirical results, and compares our algorithm to previous state-of-the-art algorithms. This section concludes with an analysis of FAQ on our motivating problem of matching worm brain connectomes. We conclude with a discussion of possible extensions of FAQ and related work in Section 5.

## 2 Preliminaries

In this section, we formally define the QAP and the GMP. We then show how the GMP can be recast as a special case of the QAP.

### 2.1 Quadratic Assignment Problems

We first define the general quadratic assignment problem. Let *A* = (*a*_*uv*_), *B* = (*b*_*uv*_) ∈ ℝ^*n*×*n*^ be two *n* × *n* real matrices. Let Π: = Π_*n*_ be the set of permutation functions (bijections) of the set [*n*] = {1,…, *n*}. We define the Koopmans-Beckmann (KB) version QAP via:

$$\begin{array}{c} \text{(KB)}\; \end{array}\begin{array}{cc} \text{minimize} & \sum_{u,v\in \left[n\right]}\;b_{uv}a_{\pi \left(u\right)\pi \left(v\right)}\\ \text{subject}\;\text{to} & \pi \in \Pi . \end{array}$$ (1)

Note that occasionally an additional linear function is added, though we drop it here for brevity.

Eq (1) can also be recast in matrix notation. Let 𝒫 be the set of *n* × *n* *permutation matrices*, $𝒫=\left\{P\in {\left\{0,1\right\}}^{n\times n}:P^{⊤}1=P1=1\right\}$, where **1** is the *n*-dimensional column vector consisting of all 1’s.

Thus, Eq (1) can be written more compactly in matrix notation via:

$$\begin{array}{c} \text{(QAP)}\;\begin{array}{cc} \text{minimize} & \text{trace}(APB^{⊤}P^{⊤})\\ \text{subject}\;\text{to} & P\in 𝒫˙ \end{array} \end{array}$$ (2)

We hereafter refer to (2) as the QAP optimization function.

### 2.2 Relaxed Quadratic Assignment Problem

Eq (2) is a binary quadratic program with linear constraints. Because of the combinatorial nature of the feasible region, finding a *global* optimum of (2) is **NP**-hard. Rather than directly optimizing over the permutation matrices, we begin by relaxing the constraint set to the convex hull of 𝒫, the set of doubly stochastic matrices (i.e. the Birkhoff polytope),

$$\begin{array}{c} 𝒟:={𝒟}_{n}=\left\{P\in {\mathbb{R}}^{n\times n}:P^{⊤}1=P1=1,P⪰0\right\}, \end{array}$$

where ⪰ indicates an element-wise inequality. Relaxing 𝒫 to 𝒟 in (2) yields the relaxed quadratic assignment problem (rQAP):

$$\begin{array}{c} \text{(rQAP)}\;\begin{array}{cc} \text{minimize} & \text{trace}(APB^{⊤}P^{⊤})\\ \text{subject}\;\text{to} & P\in 𝒟 \end{array} \end{array}.$$ (3)

Note that, although rQAP is a quadratic program with linear constraints, it is *not* necessarily convex. Indeed, the objective function, $f(P)=\text{trace}(APB^{⊤}P^{⊤})$, has a Hessian that is not necessarily positive definite: $\nabla^{2}f(P)=B⊗A+B^{⊤}⊗A^{⊤}$, where ⊗ indicates the Kronecker product (note that if *A* and *B* are hollow matrices—as is common for graphs—then ∇^2^ *f*(*P*) has trace equal to 0, and is indefinite).

While nonconvex quadratic optimization is, in general, **NP**-hard, relaxing the feasible region allows us to employ the tools of continuous optimization to search for a *local* optima of (3). These local optima can then be projected onto 𝒫, yielding an approximate solution of (2). We also note that when relaxed to 𝒟, the QAP optimization function is often multimodal, making initialization important when solving (3).

### 2.3 Graph Matching

A labeled graph *G* = (𝒱,ℰ) consists of a vertex set 𝒱 = [*n*], and an edge set $ℰ\subset \left(\begin{array}{c} 𝒱\\ 2 \end{array}\right)$ in the undirected case, or ℰ ⊂ 𝒱 × 𝒱 in the directed case. For an *n*-vertex graph *G*, we define the associated adjacency matrix *A* = (*a*_*uv*_) ∈ {0,1}^*n* × *n*^ to be the binary *n* × *n* matrix with *a*_*uv*_ = 1if {*u*, *v*} ∈ ℰ in the undirected setting, or (*u*, *v*) ∈ ℰ in the directed setting. Given a pair of *n*-vertex graphs *G*_*A*_ = (𝒱_*A*_,ℰ_*A*_) and *G*_*B*_ = (𝒱_*B*_,ℰ_*B*_), with respective adjacency matrices *A* and *B*, we consider the following two closely related problems:

- **Graph Isomorphism (GI)**: Does there exist a *P* ∈ 𝒫 such that $A=PBP^{⊤}$.
- **Graph Matching**: $\min_{P\in 𝒫}\|A-PBP^{⊤}{\|}_{F}$, where ‖·‖_*F*_ is the usual matrix Frobenius norm.

GI is one of few problems with unknown computational complexity in the **NP**-hierarchy [19]; indeed, if **P** ≠ **NP**, then GI might reside in an intermediate complexity class called **GI**-complete. Moreover, GI is, at worst, only moderately exponential, with complexity 𝒪(exp{*n* ^1/2+*o*(1)^}) [20]. On the other hand, the (harder) GMP—reducible to a QAP—is known to be **NP**-hard in general. Although polynomial time algorithms are available for GM (and GI) for large classes of problems (e.g., planar graphs, trees) [21], these algorithms often have lead constants which are very large. For example, if all vertices have degree less than *k*, there is a linear time algorithm for GI. However, the hidden constant in this algorithm is (512*k* ^3^)! [22]. Because we are interested in solving GM for graphs with $\dot{\approx }10^{6}$ or more vertices, exact GM solutions will be computationally intractable. As such, we develop a fast approximate graph matching algorithm. Our approach is based on formulating GM as a quadratic assignment problem.

### 2.4 Graph Matching as a Quadratic Assignment Problem

Given a pair of *n*-vertex graphs *G*_*A*_ = (𝒱_*A*_, ℰ_*A*_) and *G*_*B*_ = (𝒱_*B*_, ℰ_*B*_), with respective adjacency matrices *A* and *B*, we can formally write the graph matching problem as an optimization problem:

$$\begin{array}{c} \begin{array}{cc} \text{minimize} & {\left\|AP-PB\right\|}_{F}^{2}\\ \text{subject}\;\text{to} & P\in 𝒫˙ \end{array} \end{array}$$ (4)

Simple algebra yields that,

$$\begin{array}{ccc} {\left\|AP-PB\right\|}_{F}^{2} & = & \text{trace}\left\{{\left(AP-PB\right)}^{⊤}\left(AP-PB\right)\right\}\\ & = & \text{trace}\left(A^{⊤}A\right)+\text{trace}\left(BB^{⊤}\right)-2\text{trace}\left(APB^{⊤}P^{⊤}\right)\cdot \end{array}$$ (5)

The GMP is then equivalent (i.e. same argmin) to

$$\begin{array}{c} \text{(GM)}\;\begin{array}{cc} \text{minimize} & -\text{trace}(APB^{⊤}P^{⊤})\\ \text{subject}\;\text{to} & P\in 𝒫\cdot \end{array} \end{array}$$ (6)

The objective function for GM is just the negative of the objective function for QAP. Thus, any descent algorithm for the former can be directly applied to the latter. Moreover, any QAP approximation algorithms also immediately yields an analogous GM approximation.

As is common in solving general QAPs, GM algorithms often begin by first relaxing (4) to a continuous problem (see, for example, [23]). The resulting problem is a convex quadratic program and can be efficiently exactly solved. The obtained solution is then projected back onto 𝒫 yielding an approximate solution to (4). Contrary to popular existing approaches, our FAQ algorithm first solves a relaxed version of (6) and subsequently projects the solution back onto 𝒫. This relaxation yields an indefinite quadratic program, and indefinite quadratic programs are are **NP**-hard to solve in general. However, recent theory indicates that the indefinite relaxation of (6), and *not* the convex relaxation of (4) is the provably correct approach [24]. Reflecting this theory, we find that FAQ obtains state-of-the-art performance in terms of both computational efficiency and objective function value for various QAPs (see Section 4).

## 3 Fast Approximate QAP Algorithm

Our algorithm, called FAQ, proceeds in three steps:

- Choose a suitable initial position
- Find a local solution to rQAP.
- Project back onto the set of permutation matrices.

These steps are summarized in Algorithm 1. Below, we provide details for each step.

**Algorithm 1** FAQ for finding a local optimum of rQAP

**Require**: Graphs (adjacency matrices) *A* and *B* as well as a stopping criteria

**Ensure**: $\hat{P}$, an estimated permutation matrix

 1: Choose an initialization, $P^{\left(0\right)}=\text{1}{\text{1}}^{⊤}/n$

 2: **while** stopping criteria not met **do**

 3: Compute the gradient of f at the current point via Eq (3)

 4: Compute the direction *Q* ^(*i*)^ by solving Eq (8) via the Hungarian algorithm

 5: Compute the step size *α* ^(*i*)^ by solving Eq (9)

 6: Update *P* ^(*i*)^ according to Eq (10)

 7: **end while**

 8: Obtain $\hat{P}$ by solving Eq (11) via the Hungarian algorithm.

- **Find a suitable initial position**. While any doubly stochastic matrix would be a feasible initial point, we choose the noninformative “flat doubly stochastic matrix,” $J=\text{1}\cdot {\text{1}}^{⊤}/n$, i.e. the barycenter of the feasible region. Alternately, we could use multiple restarts, each initial point near *J*. Specifically, we could sample *K*, a random doubly stochastic matrix using 10 iterations of Sinkhorn balancing [25], and let *P* ^(0)^ = (*J*+*K*)/2. Given this initial estimate (or estimates), we would then iterate the following five steps until convergence.
- **Find a local solution to rQAP**. As mentioned above, rQAP is a quadratic problem with linear constraints. A number of off-the-shelf algorithms are readily available for finding local optima in such problems. We utilize the Frank-Wolfe algorithm (FW), a successive first-order optimization procedure originally devised to solve convex quadratic programs [5, 26]. Although FW is a relatively standard solver, especially as a subroutine for QAP algorithms [27], we provide a detailed view of applying FW to rQAP.
  Given an initial position, *P* ^(0)^, iterate the following four steps:
*Step 1*, *Compute the gradient ∇*f*(*P*^(*i*)^)*: The gradient of $f(P)=-\text{trace}(APB^{⊤}P^{⊤})$ with respect to *P*, evaluated at *P* ^(*i*)^, is given by $\nabla f(P^{\left(i\right)})=-AP^{\left(i\right)}B^{⊤}-A^{⊤}P^{\left(i\right)}B$.
*Step 2*, *Compute the search direction *Q*^(*i*)^*: The search direction is given by the argument that minimizes a first-order Taylor series approximation to *f*(*P*) around the current estimate, *P* ^(*i*)^:
  $$\begin{array}{c} {\tilde{f}}^{\left(i\right)}\left(P\right):=f\left(P^{\left(i\right)}\right)+\nabla f{\left(P^{\left(i\right)}\right)}^{⊤}\left(P-P^{\left(i\right)}\right)\cdot \end{array}$$ (7)
  Dropping terms independent of *P*, we obtain the following sub-problem:
  $$\begin{array}{c} \begin{array}{cc} \text{minimize} & \text{trace}(\nabla f{\left(P^{\left(i\right)}\right)}^{⊤}P)\\ \text{subject}\;\text{to} & P\in 𝒟, \end{array} \end{array}$$ (8)
  which is equivalent to a *Linear Assignment Problem* (LAP), and can therefore be solved in *O*(*n* ^3^) time via the “Hungarian Algorithm” of [28, 29]. Let *Q* ^(*i*)^ indicate the argmin of Eq (8).
*Step 3*, *Compute the step size *α*^(*i*)^*: Given *Q* ^(*i*)^, we minimize the original optimization problem, along the line segment from *P* ^(*i*)^ to *Q* ^(*i*)^:
  $$\begin{array}{c} \begin{array}{cc} \text{minimize} & f(P^{\left(i\right)}+\alpha^{\left(i\right)}Q^{\left(i\right)})\\ \text{subject}\;\text{to} & \alpha \in [0,1]\cdot \end{array} \end{array}$$ (9)
  This can be solved exactly, as *f* is a quadratic function of *α*. Let *α* ^(*i*)^ indicate the argmin of Eq (9).
*Step 4*, *Update *P*^(*i*)^*: Finally, the next iterate is the doubly stochastic matrix
  $$\begin{array}{c} P^{\left(i+1\right)}=P^{\left(i\right)}+\alpha^{\left(i\right)}Q^{\left(i\right)}\cdot \end{array}$$ (10)
*Stopping criteria*: Steps 1–4 are iterated until some stopping criterion is met. Often, a threshold *ϵ* > 0 or an iteration limit is given, and the algorithm iterates until the iteration limit is reached, ‖*P* ^(*i*)^−*P* ^(*i*−1)^‖_*F*_ < *ϵ*, or ‖∇*f*(*P* ^(*i*)^)‖_*F*_ < *ϵ*. In practice, the algorithm often converges with a modest number of FW iterates.
- **Project onto the set of permutation matrices**. Let *P* ^(*final*)^ be the doubly stochastic matrix resulting from the final iteration of FW. We project *P* ^(*final*)^ onto the set of permutation matrices via
  $$\begin{array}{c} \begin{array}{cc} \text{minimize} & -\text{trace}(P^{\left(final\right)}P^{⊤})\\ \text{subject}\;\text{to} & P\in 𝒫\cdot \end{array} \end{array}$$ (11)
  Note that Eq (11) is again equivalent to a LAP and can be solved in *O*(*n* ^3^) time.

## 4 Results

Below we present a number of empirical and theoretical results demonstrating the state-of-the-art efficiency and accuracy of the FAQ algorithm.

### 4.1 Algorithm Complexity and leading constants

**Fig 1:**
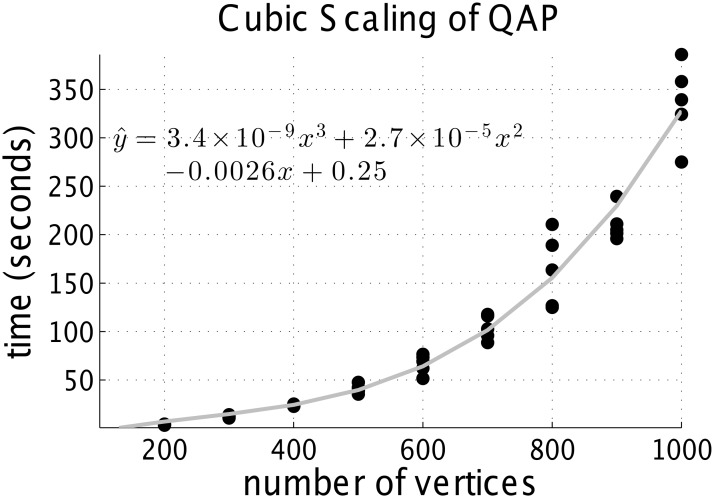
Running time of FAQ as function of number of vertices. Data was sampled from an Erdös-Rényi model with *p* = *log*(*n*)/*n*. Each dot represents a single simulation, with 100 simulations per *n*. The solid line is the best fit cubic function. Note the leading constant is ≈ 10^−9^. FAQ finds the optimal objective function value in every simulation.

As mentioned above, GM is computationally difficult, and even in the special cases for which polynomial time algorithms are available, the leading constants are intractably large. Given a bounded number of FW iterates, the FAQ algorithm has complexity *O*(*n* ^3^). However, a very large lead order constant could still render this algorithm practically infeasible. Fig 1 suggests that FAQ has *O*(*n* ^3^) complexity, and also has very small leading constants (≈ 10^−9^). This suggests that this algorithm is feasible for matching even reasonably large graphs. Note that other state-of-the-art approximate graph matching algorithms also have cubic or worse time complexity in the number of vertices. We will describe these other algorithms and their time complexity in greater detail below.

### 4.2 QAP Benchmark Accuracy

Having demonstrated both theoretically and empirically that FAQ has cubic time complexity, we evaluate the algorithms accuracy on a suite of standard benchmarks. More specifically, QAPLIB is a library of 137 quadratic assignment problems, ranging in size from 10 to 256 vertices [30]. Recent graph matching papers typically evaluate the performance of their algorithm on 16 of the benchmark QAPs that are known to be particularly difficult [23, 31]. We compare the results of FAQ to the results of four other state-of-the-art graph matching algorithms:

1. the PATH algorithm, which solves the relaxation of (4), and then solves a sequence of concave and convex problems to project the solution onto 𝒫 [23];
2. QCV which solves the relaxation of (4), and projects the obtained solution onto the closest permutation in 𝒫;
3. the RANK algorithm [32], a spectral graph matching procedure;
4. the Umeyama algorithm (denoted by U), another spectral graph matching procedure [1].

Note that the code for these four algorithms is freely available from the graphm package [23].

**Fig 2:**
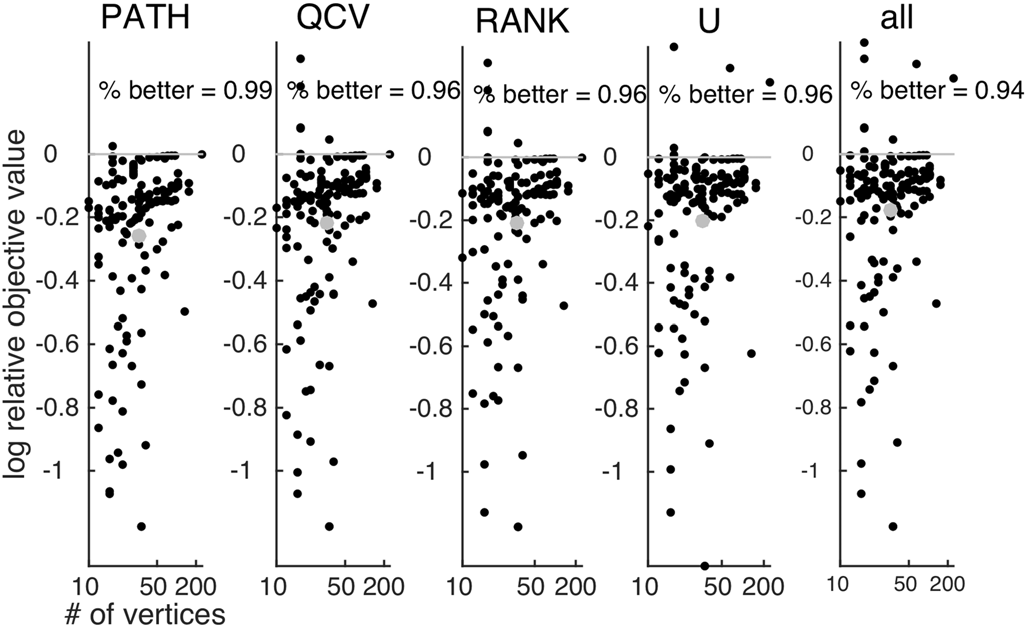
Relative accuracy—defined to be $\log_{10}({\hat{f}}_{FAQ}/{\hat{f}}_{X})$—of all the four algorithms compared with FAQ. Note that FAQ is better than all the other algorithms on ≈ 94% of the benchmarks. The abscissa is the log number of vertices. The gray dot indicates the mean improvement of FAQ over the other algorithms.

Performance is measured by minimizing the assignment cost $f(P)=\text{trace}(APB^{⊤}P^{⊤})$. We write ${\hat{f}}_{X}$ for the value of the local minimum of *f* obtained by the algorithm *X* ∈ {FAQ, PATH, QCV, RANK, U, all}, where “all” is just the best performer of all the non FAQ algorithms. Fig 2 plots the logarithm (base 10, here and elsewhere) of the relative accuracy, i.e. $\log_{10}({\hat{f}}_{F\mathrm{AQ}}/{\hat{f}}_{X})$, for *X* ∈ {PATH, QCV, RANK, U, all}. FAQ performs significantly better than the other algorithms, outperforming all of them on ≈ 94% of the problems, often by nearly an order of magnitude in terms of relative error.

### 4.3 QAP Benchmark Efficiency

**Fig 3:**
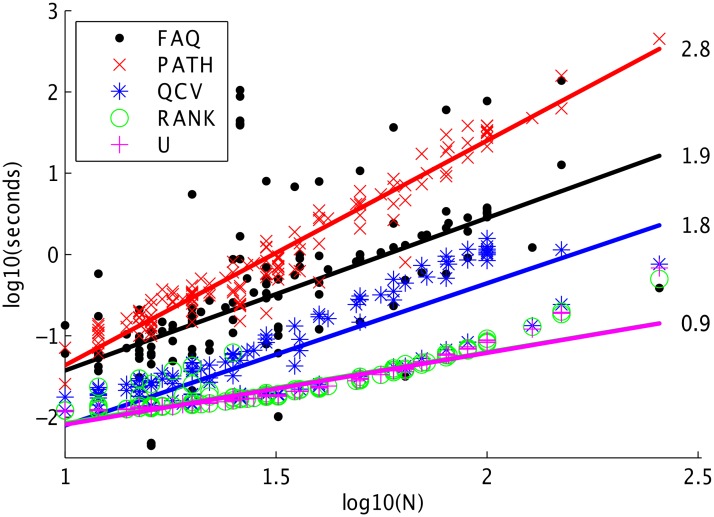
Absolute wall time for running each of the five algorithms on all 137 QAPLIB benchmarks. We fit a line on this log-log plot for each algorithm; the slope is displayed beside each line. The FAQ slope is much better than the PATH slope, and worse than the others. Note, however, the time for RANK and U appears to be superlinear on this log-log plot, suggesting that perhaps as the number of vertices increases, PATH might be faster.

The utility of an approximation algorithm depends not just on its accuracy, but also its efficiency. To empirically test these algorithms’ efficiency, we compare the wall time of each of the five algorithms on all 137 QAPS in QAPLIB in Fig 3. For each of the 5 algorithms, we fit an iteratively weighted least squares linear regression function (Matlab’s “robustfit”) to regress the logarithm of time (in seconds) onto the logarithm of the number of vertices being matched. The numbers beside the lines indicate the slopes of the regression functions.

The figure demonstrates that the PATH algorithm has the largest slope, significantly larger than that of FAQ. QCV and FAQ have nearly identical slopes, which makes sense, given that the are solving very similar objective functions. Similarly, RANK and U have very similar slopes; they are both using spectral approaches. Note, however, that although the slope of RANK and U are smaller than that of FAQ, they both appear to be super linear in this log-log plot, suggesting that as the number of vertices increases, their compute time might exceed that of the other algorithms.

Combined with Fig 2, this figure suggests that FAQ achieves the state-of-the-art trade-off between efficiency and accuracy. Of note is that the FAQ algorithm has a relatively high variance in wall time for these problems. This is due to the number of Hungarian algorithms performed in the FW subroutine. We could fix the number of Hungarian algorithms, in which case the variance would decrease dramatically. However, in application, this variance is not problematic.

### 4.4 QAP Benchmark Accuracy/Efficiency Trade-off

**Fig 4:**
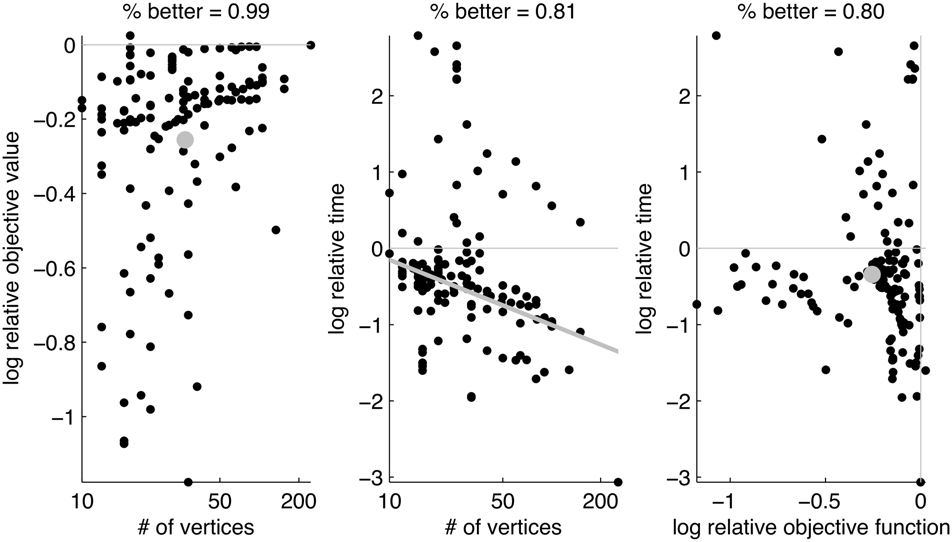
Comparison of FAQ with PATH in terms of both accuracy and efficiency. The left panel is the same as the left panel of Fig 2. The middle plots the relative wall time of FAQ to PATH as a function of the number of vertices, also on a log-log scale. The gray line is the best fit slope on this plot. Finally, the right panel plots log relative time versus log relative objective function value, demonstrating that FAQ outperforms PATH on both dimensions on 80% of the benchmarks.

In [23], the authors demonstrated that PATH outperformed both QCV and U on a variety of simulated and real examples. Fig 4 compares the performance of FAQ with PATH along both dimensions of performance—accuracy and efficiency—for all 137 benchmarks in the QAPLIB library. The right panel indicates that FAQ is *both* more accurate and more efficient on 80% of the problems (and is more accurate on 99% of the benchmarks). The middle plots the relative wall time of FAQ to PATH as a function of the number of vertices, also on a log-log scale. The gray line is the best fit slope on this plot, suggesting that FAQ is getting exponentially faster than PATH as the number of vertices gets larger.

### 4.5 QAP Directed Benchmarks

Recently, Liu et al. [33] proposed a modification of the PATH algorithm that adjusted PATH to be more appropriate for directed graphs Note that our FAQ algorithm easily extend to directed or weighted graphs. Liu et al. compare the performance of their algorithm (EPATH) with U, QCV, and the GRAD algorithm of [34] on a set of 16 particularly difficult directed benchmarks from QAPLIB. The EPATH algorithm achieves at least as low objective value as the other algorithms on 15 of 16 benchmarks. Our algorithm, FAQ, performs at least as well as EPATH, U, QCV, and GRAD on all 16 benchmarks, and achieves the singular best performance on 8 of the benchmarks. Table 1 shows the numerical results comparing FAQ to EPATH and GRAD, the only algorithm considered in [33] to outperform EPATH. Note that some of the algorithms achieve the absolute minimum on 8 of the benchmark.

**Table 1:** Comparison of FAQ with optimal objective function value and previous state-of-the-art for directed graphs. The best (lowest) value is in **bold**. Asterisks indicate achievement of the global minimum. The number of vertices for each problem is the number in its name (second column).

#	Problem	Optimal	FAQ	EPATH	GRAD
1	lipa20a	3683	**3791**	3885	3909
2	lipa20b	27076	27076*	32081	27076*
3	lipa30a	13178	**13571**	13577	13668
4	lipa30b	151426	151426*	151426*	151426*
5	lipa40a	31538	**32109**	32247	32590
6	lipa40b	476581	476581*	476581*	476581*
7	lipa50a	62093	**62962**	63339	63730
8	lipa50b	1210244	1210244*	1210244*	1210244*
9	lipa60a	107218	**108488**	109168	109809
10	lipa60b	2520135	2520135*	2520135*	2520135*
11	lipa70a	169755	**171820**	172200	173172
12	lipa70b	4603200	4603200*	4603200*	4603200*
13	lipa80a	253195	**256073**	256601	258218
14	lipa80b	7763962	7763962*	7763962*	7763962*
15	lipa90a	360630	**363937**	365233	366743
16	lipa90b	12490441	12490441*	12490441*	12490441*

### 4.6 Theoretical properties of FAQ

In addition to guarantees on computational time, we have a guarantee on performance:

**Proposition 1** *If A is the adjacency matrix of an asymmetric simple graph G, then*

$$\underset{{}_{D\in 𝒟}}{\text{argmin}}\begin{array}{c} -trace\left(ADA^{⊤}D^{⊤}\right)=\left\{I\right\}. \end{array}$$

*Proof* Let *m* denote the number of edges in *G*. As *G* is asymmetric,

$$\begin{array}{c} \text{trace}\left(APA^{⊤}P^{⊤}\right)<2m=\text{trace}\left(AA^{⊤}\right) \end{array}$$

for any *P* ≠ *I*. By the Birkhoff-von Neuman Theorem, 𝒟 is the convex hull of 𝒫, i.e., for all *D* ∈ 𝒟, there exists constants {*α*_*D*, *P*_}_*P* ∈ 𝒫_ such that *D* = ∑_*P* ∈ 𝒫_ *α*_*D*, *P*_ *P* and ∑_*P* ∈ 𝒫_ *α*_*D*, *P*_ = 1. Thus, if *D* is not the identity matrix,

$$\begin{array}{ccc} \text{trace}(ADA^{⊤}D^{⊤}) & = & \sum_{P}\sum_{Q}\alpha_{D,P}\alpha_{D,Q}\text{trace}\left(APA^{⊤}Q^{⊤}\right)\\ & = & \sum_{P}\alpha_{D,P}^{2}\underset{<2m\;\text{if}\;P\neq I}{\underset{︸}{\text{trace}(APA^{⊤}P^{⊤})}}+\sum_{P}\sum_{Q\neq P}\alpha_{D,P}\alpha_{D,Q}\underset{\leq 2m}{\underset{︸}{\text{trace}(APA^{⊤}Q^{⊤})}}\\ & < & 2m \end{array}$$

as *D* ≠ *I* implies that *α*_*D*, *P*_ > 0 for some *P* ≠ *I*. ■

**Remark 1** Note that it trivially follows from Proposition 1 that if A and B are the adjacency matrices of asymmetric isomorphic simple graphs, then the minimum objective function value of rGMP is equal to the minimum objective function value of GMP.

**Remark 2** For the convex quadratic GM relaxation, Eq (4), in general ${\text{argmin}}_{D\in 𝒟}\|AD-DA{\|}_{F}^{2}\neq \{I\},$ *even if *G* (the graph with adjacency matrix *A*) is asymmetric. Indeed, degree regular graphs provide a simple counterexample, as in this case* $J\in {\text{argmin}}_{D\in 𝒟}\|AD-DA{\|}_{F}^{2}$. We will empirically explore the ramifications of this phenomena further in Section 4.8

This result says that, when solving the GI problem, nothing is lost by relaxing the indefinite GM problem as done by FAQ. Recent results also show this is almost surely the case (in a broad class of random graphs) when relaxing the indefinite GM problem, even in the non-isomorphic graph setting [24] (and is again almost surely *not* the case when relaxing the convex GM formulation). Combined, this serve to provide theoretical justification for our FAQ procedure.

### 4.7 Multiple Restarts

Due to the indefiniteness of the relaxation of (6), the feasible region may be rife with local minima. As a result, our FAQ procedure is sensitive to the chosen initial position. With this in mind, we propose a variant of the FAQ algorithm in which we run the FAQ procedure with multiple initializations. The algorithm outputs the best FAQ iterate over all the initializations. For each initialization, we sample *K* ∈ 𝒟, a random doubly stochastic matrix, using 10 iterations of Sinkhorn balancing [25], and let our initialization be *P* ^(0)^ = (*J*+*K*)/2, where *J* is the doubly stochastic barycenter. Fixing the number of restarts, this variant of FAQ still has *O*(*n* ^3^) complexity.

Table 2 shows the performance of running FAQ 3 and 100 times, reporting only the best result (indicated by FAQ_3_ and FAQ_100_, respectively), and comparing it to the best performing result of the five algorithms (including the original FAQ)—note that the best performing of the original five tested was always FAQ. Note that we only consider the 16 particularly difficult benchmarks for this evaluation. FAQ only required three restarts to outperform all other approximate algorithms on all 16 of 16 difficult benchmarks. Moreover, after 100 restarts, FAQ finds the absolute minimum on 3 of the 16 benchmarks. Note that no other algorithm ever achieved the absolute minimum on any of these benchmarks.

**Table 2:** Comparison of FAQ with optimal objective function value and the best result on the undirected benchmarks. Note that FAQ restarted 100 times finds the optimal objective function value in 3 of 16 benchmarks, and that FAQ restarted 3 times finds a minimum better than the previous state-of-the-art on all 16 particularly difficult benchmarks.

#	Problem	Optimal	FAQ_100_	FAQ_3_	FAQ_1_
1	chr12c	11156	**12176**	13072	13072
2	chr15a	9896	9896*	17272	19086
3	chr15c	9504	**10960**	14274	16206
4	chr20b	2298	**2786**	3068	3068
5	chr22b	6194	**7218**	7876	8482
6	esc16b	292	292*	294	296
7	rou12	235528	235528*	238134	253684
8	rou15	354210	**356654**	371458	371458
9	rou20	725522	**730614**	743884	743884
10	tai10a	135028	**135828**	148970	152534
11	tai15a	388214	**391522**	397376	397376
12	tai17a	491812	**496598**	511574	529134
13	tai20a	703482	**711840**	721540	734276
14	tai30a	1818146	**1844636**	1890738	1894640
15	tai35a	2422002	**2454292**	2460940	2460940
16	tai40a	3139370	**3187738**	3194826	3227612

**Remark 3** Another natural starting position for FAQ is the doubly stochastic solution of rGMP, the relaxed Eq (4). Promising results in this direction are pursued further in [24].

### 4.8 Brain-Graph Matching

The *Caenorhabditis elegans* (*C. elegans*) is a small worm (nematode) with 302 labeled neurons (in the hermaphroditic sex). The chemical connectome of *C. elegans* is a weighted directed graph on 279 vertices, with edge weights in {0,1,2,…} counting the number of directed chemical synapses between the neurons [35, 36]. We conduct the following synthetic experiments. For *A* = (*A*_*uv*_) count the number of synapses (in {0,1,2,…}) from neuron *u* to neuron *v* in the C. elegans connectome, for all *u*, *v*. For *k* = 1,2,…,1000, we choose *Q*_(*k*)_ uniformly at random from 𝒫, and let $B_{\left(k\right)}=Q_{\left(k\right)}A{Q_{\left(k\right)}}^{⊤}$, effectively shuffling the vertex labels of the connectome. Then, we match graphs *A* to *B*_(*k*)_. We define accuracy as the fraction of vertices correctly assigned (i.e. unshuffled).

**Fig 5:**
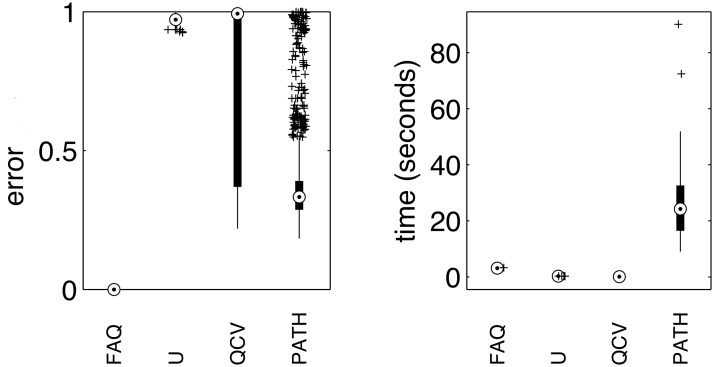
Performance of U, QCV, PATH, and FAQ on synthetic C. elegans connectome data, graph matching the true chemical connectome with permuted versions of itself. Error is the fraction of vertices incorrectly matched. Circle indicates the median, thick black bars indicate the quartiles, thin black lines indicate extreme but non-outlier points, and plus signs are outliers. The left panel indicate error (fraction of misassigned vertices), and the right panel indicates wall time on a 2.2 GHz Apple MacBook. FAQ always obtained the optimal solution, whereas none of the other algorithms ever found the optimal. FAQ also ran very quickly, nearly as quickly as U and QCV, and much faster than PATH, even though the FAQ implementation is in Matlab, and the others are in C.

Fig 5 displays the results of FAQ (initialized at *J*) along with U, QCV, and PATH. The left panel indicates that FAQ *always* perfectly unshuffles the chemical connectome, whereas none of the other algorithms ever perfectly unshuffles the graph. In light of Proposition 1, this is unsurprising. Indeed, there is a unique automorphism for this connectome, and the graph is asymmetric. For any choice of *Q*_(*k*)_, the indefinite problem (6) therefore has a unique solution, namely $Q_{\left(k\right)}^{⊤}$. FAQ finds this global minima in all the cases. Contrast this with Eq (4)—the objective function of PATH and QCV—which could have multiple global minima in 𝒟. This could account for the high variance in the performance of QCV and PATH in Fig 5.

The right panel compares the wall time of the various algorithms, running on an 2.2 GHz Apple MacBook. Note that we only have a Matlab implementation of FAQ, whereas the other algorithms are implemented in C. Unlike in the QAPLIB benchmarks, FAQ runs nearly as quickly as both U and QCV; and as expected, FAQ runs significantly faster than PATH. We also ran FAQ on a binarized symmeterized versions of the chemical connectome graph $\tilde{A}$ (i.e. ${\tilde{A}}_{uv}=1$ if and only if *A*_*uv*_ ≥ 1 or *A*_*vu*_ ≥ 1). The resulting errors are nearly identical to those presented in Fig 5, although speed increased for FAQ by more than a factor of two. Note that the number of vertices in this brain-graph matching problem—279—is larger than the largest of the 137 benchmarks used above.

## 5 Discussion

This work presents the FAQ algorithm, a fast algorithm for approximately matching very large graphs. Our key insight was to continuously relax the *indefinite* formulation of the GMP in the FW implementation. We demonstrated that not only is FAQ cubic in time, but also its leading constant is quite small—10^−9^—suggesting that it can be used for graphs with hundreds or thousands of vertices (§4.1).

Moreover, FAQ achieves better accuracy than previous state-of-the-art approximate algorithms on on over 93% of the 137 QAPLIB benchmarks (§4.2), is faster than the state-of-the-art PATH algorithm (§4.3), and is both faster and achieves at least as low performance as PATH on over 80% of the tested benchmarks (§4.4), including both directed and undirected graph matching problems (§4.5). In addition to the theoretical guarantees of cubic run time, we provide theoretical justification for optimizing the indefinite GM formulation, Eq (6) as opposed to Eq (4) (§4.6). Indeed, the indefinite formulation (and *not* the convex formulation) has the property that when matching asymmetric isomorphic graphs, the unique global minimum of the indefinite relaxation is the isomorphism between the two graphs.

Because rQAP is indefinite, we also considered FAQ with multiple restarts, and achieve improved performance for the particularly difficult benchmarks using only three restarts (§4.7). Finally, we used FAQ to match permuted versions of the C. elegans connectomes (§4.8). Of the four state-of-the-art algorithms considered, FAQ achieved perfect performance 100% of the time, whereas none of the other three algorithms ever matched the connectomes perfectly. Moreover, FAQ ran comparably fast to U and QCV and significantly faster than PATH, even though FAQ is implemented in Matlab, and the others are implemented in C. Note that these connectomes have 279 vertices, more vertices than the largest QAP benchmarks.

### 5.1 Related Work

Our approach is quite similar to other approaches that have recently appeared in the literature. Perhaps its closest cousins include [23, 37–39], which are all of the “PATH” following or FW varieties. These algorithms begin by relaxing the convex objective function in (4), while FAQ begins by relaxing the indefinite objective function in (6). Although the convex relaxation is efficiently solvable, the obtained solution is almost surely incorrect (for a broad class of random graphs) and the correct solution is often not obtained even post projection [24]. The indefinite relaxation however, almost surely yields the correct solution when exactly solved (for a broad class of random graphs) [24]. With this in mind, it is unsurprising that FAQ outperforms PATH on nearly all benchmark problems. Others have considered similar relaxations to PATH, but usually in the context of finding lower bounds [40] or as subroutines for finding exact solutions [41]. Our work seems to be the first to utilize the precise algorithm described in Algorithm 1 to find fast approximate solutions to GMP and QAP.

We note that the work in [38] is a generalization of our FW approach. The authors of [38] apply FW with exact line search to find

$$\begin{array}{c} x^{*}=\text{argmax}\left(x^{⊤}Mx\right)\;\text{s.t.}\;Cx=1,x\in {\left\{0,1\right\}}^{n}, \end{array}$$ (12)

where *M* is an *n* ^2^ × *n* ^2^ matrix whose *M*_*i*, *j*;*k*,ℓ_ entry measures the similarity between the vertex pairs (*i*, *k*) and (*j*,ℓ), and *C* is a constraint matrix enforcing the one–to–one (easily generalizing to many–to–one) matching of vertices. Our GM formulation (6), and our subsequent FW implementation, can be realized from (12) by setting *M* = −*B* ^*T*^ ⊗ *A* ^*T*^, and having *C* enforce a one–to–one matching of the vertices. Our approach offers several distinct advantages over the general approach of [38], namely:

- The ability to efficiently employ multiple random restarts for enhanced performance (see Section 4.7 for detail);
- Our FW implementation exploits the Kronecker product structure of *M* which reduces the runtime of an *O*(*n* ^4^) procedure to *O*(*n* ^3^);
- Our final step is projected to the nearest permutation matrix, which is not part of the algorithm outlined in [38];
- In [38], they consider general *M*, and in so doing, they are considering all quadratic-objective optimizations (albeit without a linear and constant term). There are many quadratic formulations of GM, and our contribution is touting the efficiency and efficacy of solving our particular indefinite form.

### 5.2 Future Work

Even with the very small lead order constant for FAQ, as *n* increases, the computational burden gets quite high. For example, extrapolating the curve of Fig 1, this algorithm would take about 20 years to finish (on a standard laptop) when *n* = 100,000. We hope to be able to approximately solve rQAP on graphs much larger than that, given that the number of neurons even in a fly brain, for example, is ≈ 250,000. More efficient algorithms and/or implementations are required for such massive graph matching. Although a few other state-of-the-art algorithms were more efficient than FAQ, their accuracy was significantly worse. We are actively working on combining FAQ with dimensionality reduction procedures to achieve the desired scaling from FAQ [42].

We are also pursuing additional future work to generalize FAQ in a number of ways:

- In addition to the theoretical results contained in Section 4.6, we have studied the properties of the convex and indefinite GMP relaxations in a very general random graph model [24]. Under some general assumptions on the random graph model, the indefinite relaxation of (6), and *not* the convex relaxation of (4) is the provably correct approach [24].
- Many (brain-) graphs of interest will be errorfully observed [43], that is, vertices might be missing and putative edges might exhibit both false positives and negatives. Explicitly dealing with this error source is both theoretically and practically of interest [15].
- For many brain-graph matching problems, the number of vertices will not be the same across the brains. Recent work from [23, 37] and [39] suggest that extensions in this direction would be both relatively straightforward and effective.
- Often, a partial matching of the vertices is known a priori, and we can modify FAQ to leverage these seeded vertices to match the remaining unseeded vertices [44].
- The most costly subroutine in FAQ is solving LAPs. Fortunately, the LAP is a linear optimization problem with linear constraints. As a result, a number of parallelized optimization strategies could be implemented on this problem [45].
- Often, real data adjacency matrices have certain special properties, namely sparsity, which makes faster LAP subroutines [29] and more efficient algorithms (such as “active set” algorithms) readily available for further speed increases.
- In many graph settings, we have some prior information that could easily be incorporated into the GM problem in the form of vertex attributes and features. For example, in brain graphs we know the position of the vertex in the brain, the vertex’s cell type, etc. These could be used to measure a “dissimilarity” between vertices and are easily incorporated into FAQ’s objective function to better match the graphs.
- We are working to extend FAQ to match multiple graphs simultaneously.

### 5.3 Concluding Thoughts

In conclusion, this manuscript has presented the FAQ algorithm for approximately solving the quadratic assignment problem. FAQ is theoretically justified, fast, effective, and easily generalizable. Our algorithm achieves state-of-the-art matching performance and efficiency on a host of benchmark QAP problems and connectome data sets. Yet, the 𝒪(*n* ^3^) complexity remains too slow to solve many problems of interest, and issues of scalability need be addressed. To facilitate further development and applications, all the code and data used in this manuscript is available from the first author’s website, http://jovo.me. We have further incorporated FAQ (as sgm.R) into the open-source R package, igraph, available for download at https://github.com/igraph/xdata-igraph/. MATLAB code is also available at https://github.com/jovo/FastApproximateQAP/tree/master/code/FAQ.
